# 

**Published:** 1832-10

**Authors:** 


### MONTHLY LIST OF MEDICAL BOOKS.

[*Medical Works cannot be entered on this List unless a copy be sent for the purpose, the titles of Books having frequently been sent to us as published which have not been published for weeks, or even months, after.*]

The Cyclopædia of Practical Medicine. Edited by John Forbes, m.d., A. Tweedie, m.d., and John Conolly, m.d. Part IX. (published in Monthly Parts,) September 1832,) Containing *Puerperal Fever,* by Dr. Lee; *Yellow Fever,* Dr. Gillkrest; *Fungus Hæmatodes,* Dr. Kerr; *Galvanism,* Dr. Apjohn; *Gastritis,* Dr. Stokes; *Gastrodynia,* Dr. Barlow; *Gastro-enteritis,* Dr. Stokes; *Glossitis,* Dr. Kerr; *Glottis, Spasm of,* Dr. Joy; *Gout,* Dr. Barlow; *Headach,* Dr. Burder.—Royal 8vo. pp. 127. Sherwood, Gilbert, and Piper; and Baldwin and Cradock, London.

A Letter addressed to the Central Board of Health, written with the View of establishing rational Principles for the Treatment of Cholera, and shewing the Danger of the Mode of Practice at present generally followed. By John George French, Surgeon to St. James's Cholera Hospital, &c.—8vo. pp. 22. Rivington, London.

Part XI. The Principles and Practice of Obstetric Medicine, in a Series of Systematic Dissertations on Midwifery and on the Diseases of Women and Children. Illustrated by numerous Plates. By D. D. Davis, m.d., m.r.s.l., &c.—Taylor, London.

On Hybernation. By Marshall Hall, m.d., f.r.s. l. and e. &c. From the Philosophical Transactions.—4to. pp. 26.

Theory of the Inverse Ratio which subsists between the Respiration and Irritability, in the Animal Kingdom. By Marshall Hall, m.d., f.r.s. l. and e. &c. From the Philosophical Transactions.—4to. pp. 14.

Documens recueillis par MM. Chervin, Louis, et Trousseau, Membres de la Commission Médicale Française envoyée à Gibraltar, pour observer l'Epidémie de 1828; et par M. le Dr. Barry, Médecin des Armées Anglaises. Tome II.—8vo. pp. 412 and 403. Paris, de l'Imprimerie Royale.

### APOTHECARIES' HALL.

NAMES OF GENTLEMEN to whom the Court of Examiners have granted Certificates:

AUGUST 30.

Joseph Boyer, of Quorndon
William Sturton, Huntingdon.

SEPT. 6.

John Burrows, of Appleton, Cheshire
Charles William Hoyland, London
James Taylor Lamb, Manchester
John Robertson, Glasgow
Robert Topping, Devonport

SEPT. 13.

George Daysh Austin, of Chatham
Leonard Morse Goddard, Shaftsbury
John Skill, London
Henry Wilkinson, Sheffield.

SEPT. 20.

Bernard Brown.
David Durran, Enfield
Richard Elgie.
Frederick Edmonds, Penzance
Samuel Pearce, Bethnal Green.

SEPT. 28.

William Pellew Bain, of London
Samuel Devenish, Ilminster
Charles Porter, Birmingham
Renforth Thomas Scarr, Bishop's Stortford

